# The acute exercise response of peripheral blood mononuclear cells and their bioenergetic function in women with high and low systemic estradiol levels

**DOI:** 10.14814/phy2.70296

**Published:** 2025-05-01

**Authors:** Sira Karvinen, Emilia Lähteenmäki, Bettina Hutz, Hanna‐Kaarina Juppi, Jari E. Karppinen, Anna Kankaanpää, Maarit Lehti, Eija K. Laakkonen

**Affiliations:** ^1^ Gerontology Research Center and Faculty of Sport and Health Sciences University of Jyväskylä Jyväskylä Finland; ^2^ Faculty of Sport and Health Sciences University of Jyväskylä Jyväskylä Finland

**Keywords:** menopause, mitochondria, white blood cell

## Abstract

Decrease in the systemic estradiol (E2) levels caused by menopause has been associated with an increased risk for cardiovascular disease. We have previously shown that E2 level is associated with the systemic response to an acute bout of endurance exercise. However, the association of systemic E2 level with peripheral blood mononuclear cell (PBMC) bioenergetic function has not been investigated. We examined the associations of systemic E2 level (HIGH and LOW E2 groups) on WBC count and PBMC bioenergetic function before and after an acute bout of endurance exercise (time points PRE, POST and 1 h POST exercise). Exercise stimulus was a maximal incremental bicycle ergometer test. We show that an acute bout of exercise induced a transient increase in WBC count in both HIGH and LOW E2 study groups (*p* < 0.001). We also observed an increase in the percentage of neutrophils and a decrease in the percentage of lymphocytes in response to exercise (*p* < 0.001). An acute bout of exercise was also associated with a transient increase in PBMC maximal electron transfer capacity and spare capacity (*p* < 0.001). No statistically significant associations were observed between systemic E2 level and PBMC bioenergetic function at the basal state or in the responses to acute exercise.

## 1 INTRODUCTION

Menopause and the accompanying changes in the systemic hormonal levels (e.g., decrease in the estradiol [E2] levels) have been associated with unfavorable changes in metabolic health (Carr, 2003; Hyvärinen et al., 2021; Karvinen et al., 2019). For example, menopause increases the risk for cardiovascular disease (CVD) (Howard et al., 2005; Karvinen et al., 2019; Pansini et al., 1993). Increasing evidence supports a role of inflammation in the atherosclerotic process (Ross, 1999; Tracy, 1998). Interestingly, several studies have established that elevated white blood cell (WBC) count increases the risk of future cardiovascular events and mortality, even in individuals without apparent CVD (Brown et al., 2001; Friedman et al., 1974; Lee et al., 2001) as well as in postmenopausal women (Margolis et al., 2005).

WBC count and composition are suggested to be indicators of the inflammatory and immune status of an individual (Chen et al., 2016). We have previously reported that postmenopausal women have lower total WBC count, lower percentage of neutrophils, and higher percentage of lymphocytes compared with pre‐ and perimenopausal women (Kovanen et al., 2018). Importantly, E2 levels were observed to be associated with the WBC count as well as the neutrophil‐to‐lymphocyte ratio, indicating that the menopause‐associated changes in WBC composition may serve as an early sign of low‐grade inflammation (Kovanen et al., 2018). Similar findings have also been observed in other menopausal cohorts (Chen et al., 2016).

In addition to the WBC count, the mitochondrial function of WBCs may contribute to inflammation (Hernández‐Aguilera et al., 2013). More specifically, peripheral blood mononuclear cells (PBMCs), consisting of lymphocytes, monocytes, and dendritic cells, are a group of immune cells that exhibit important roles in immune responses (Ulmer et al., 1984). Furthermore, PBMCs have been suggested to act as sensors for metabolic stress and serve as biomarkers of mitochondrial dysfunction in human pathologies such as type 2 diabetes and CVD (Kramer et al., 2014). Previously, Silaidos et al. (2018) showed sex‐associated differences in PBMC mitochondrial function, with females having higher mitochondrial complexes I, I + II, and IV, uncoupled respiration, and electron transport system capacity (ET‐capacity) compared to males (Silaidos et al., 2018). However, to our knowledge, the role of systemic E2 level in PBMC bioenergetic function has not been previously investigated.

Regular exercise is known to counteract low‐grade systemic inflammation (Burini et al., 2020). Endurance exercise elevates the number of PBMCs in the circulation immediately after an acute bout of exercise, affecting pro‐ and anti‐inflammatory responses (Connolly et al., 2004; Spielmann et al., 2014). More recent evidence suggests that some of the beneficial effects of regular exercise on the immune system are likely mediated by alterations in PBMC bioenergetics (Nieman & Pence, 2020; Rosa‐Neto et al., 2022). Yet, we currently lack the knowledge on whether menopause affects PBMC bioenergetics in response to an acute bout of exercise.

In the present study, we examined changes in WBC count and type as well as their bioenergetic function in response to an acute bout of endurance exercise. We also compared the associations of systemic E2 level (HIGH and LOW E2 groups) in PBMC characteristics before and after an acute bout of endurance exercise. We show that an acute bout of exercise induced a transient increase in WBC count in both HIGH and LOW E2 study groups. There was also an increase in the percentage of neutrophils and a decrease in the percentage of lymphocytes in response to exercise. The combined relative percentage of monocytes, basophils, and eosinophils decreased in response to exercise. We observed no associations between systemic E2 level and PBMC bioenergetic function at the basal state or in the responses to acute exercise.

## 2 MATERIALS AND METHODS

### 2.1 Background data

The participants of this study are derived from the Metabolism substudy (*n* = 42) of the larger Estrogen, MicroRNAs and the Risk of Metabolic Dysfunction (EsmiRs) study (Hyvärinen et al., 2021). For the present study, 37 women were included, of which 16 were classified into the HIGH E2 group and 21 into the LOW E2 group according to their systemic estradiol (E2) level (Table 1). The exclusion criteria were the following: body mass index <18 or >30 kg/m2, oophorectomy or hysterectomy, disease or medication use affecting metabolism, hormonal contraception, and regular smoking. Five women were excluded due to insufficient blood samples. The HIGH E2 group consisted of pre‐ and perimenopausal women, and women using estrogen‐based hormonal therapy, while the LOW E2 group contained postmenopausal women not using hormonal therapy (Karppinen et al., 2022). Menopausal status was assessed by measuring serum follicle‐stimulating hormone (FSH).

**TABLE 1:** Characteristics of the study participants.

Parameter	HIGH E2	LOW E2	*p* value
Age	*n* = 16	*n* = 19–21	0.385
	55.2 (1.4)	55.7 (1.7)	
BMI (body mass index)	25.0 (3.0)	24.6 (2.3)	0.705
Body fat %	37.8 (4.7)	36.3 (5.9)	0.403
VO_2_peak/body mass	28.3 (4.5)	32.3 (5.4)	**0.023**
VO_2_peak/lean body mass	47.4 (7.3)	52.7 (7.7)	**0.043**
Self‐reported leisure‐time physical activity (MET‐h/d)	5.1 (4.3)	4.8 (3.2)	0.927
Physical activity measured with an accelerometer (mg)	46.4 (28.0)	49.2 (24.2)	0.736
E2 (estradiol)	0.30 (0.18)	0.07 (0.03)	**<0.001**
FSH (follicle‐stimulating hormone)	35.8 (25.0)	88.3 (35.1)	**<0.001**

*Note*: Values show mean with standard deviation (*SD*). Values with *p* < 0.050 are marked in bold.

### 2.2 Anthropometrics and body composition

Anthropometrics, body composition, and physical fitness were measured between 7:00 and 10:00 a.m. after overnight fasting, similarly as reported previously (Kovanen et al., 2018). For determining body mass index (BMI), body weight and height were measured with the participant wearing only undergarments, and BMI was calculated as weight (kg)/height squared (m^2^). Body fat percentage and lean body mass were assessed with dual‐energy x‐ray absorptiometry (DXA) (LUNAR Prodigy; GE Healthcare, Chicago, IL).

### 2.3 Physical activity

Accelerometer‐measured physical activity was assessed with mean amplitude deviations of hip‐worn accelerometer data as described before. Briefly, participants wore accelerometers (ActiGraph GT3X and wGT3X) for 7 days during waking hours, excluding water activities. Data were collected at 60 Hz, and the Euclidian norm of acceleration was calculated. Mean amplitude deviation values were computed for 5 s epochs and expressed as their mean value (Hyvärinen et al., 2021).

Self‐reported physical activity was assessed using a questionnaire on the frequency, intensity, and duration of leisure and commuting activities. Leisure‐time physical activity in MET hours per day was then calculated from these responses (Kujala et al., 1998).

### 2.4 Hormone measurements

Systemic hormone levels were measured as reported previously (Juppi et al., 2022). Briefly, fasting serum samples were taken from the antecubital vein between 7 and 10 a.m. E2 and FSH were measured with IMMULITE 2000 XPi (Siemens Healthcare Diagnostics).

### 2.5 V̇O_2peak_ test

V̇O_2peak_ was measured with a V_max_ Encore 92 metabolic cart (Sensormedics) during a maximal incremental bicycle ergometer (Ergoselect 200, Ergoline GmbH) test, as described previously (Karppinen et al., 2022; Karvinen et al., 2023). Briefly, testing was performed after overnight fasting, and study subjects were instructed to abstain from alcohol intake, as well as strenuous exercise, for 48 h before the test. The test comprised submaximal and maximal phases. Study subjects began by cycling for 4 min at an intensity of 20 watts (W), after which the workload was increased by 20 W every 4 min until a respiratory exchange ratio of 1.0 was reached. Thereafter, the intensity was increased by 1 W/3 s to a total of 20 W/min. The test continued until volitional exhaustion. After the test, the participants performed a 5 min cooldown at an intensity of 50 W. Gas exchange was recorded as 10 s rolling averages, and the highest continuous 30 s V̇O_2_ period was selected to represent participants' absolute V̇O_2peak_. Respiratory gas exchange measurement was unreliable in three participants due to metabolic cart failure or mask‐wearing difficulties. For these participants, V̇O_2peak_ was determined based on maximal workload, using the equation created by Storer et al. (1990). V̇O_2peak_ was also scaled relative to body weight (ml/kg/min) and lean body mass (ml/kg LBM/min).

### 2.6 Blood collecting and analysis

After overnight fasting, blood samples (18 mL) were drawn from the antecubital vein of the subjects at each of the three time points (PRE, POST and 1 h POST exercise) into Vacutainer Vacuette® EDTA K3 tubes. Complete blood count (CBC) was obtained with a hematology automated analyzer (XP‐300, Sysmex). The effect of exercise‐induced plasma volume shift on WBC was calculated based on hemoglobin and hematocrit according to the modified equation by Dill & Costill (1974); (Matomäki et al., 2018). Both WBC count and PBMC bioenergetic function were measured from the same blood sample.

### 2.7 Differential white blood cell (WBC) count from stained blood samples

Two peripheral blood smears per test subject and per time point (PRE, POST, 1 h POST) were prepared from peripheral blood collected for WBC measurements. After air‐drying and methanol fixation, the blood smears were double‐stained with May Grünwald–Giemsa (Merck–Millipore) as described by the manufacturer. The stained blood smears were evaluated under a bright field microscope at 10 × magnification, and the WBCs were stained and counted manually (Sanders & Bennett, 1950). For Figure 2, the average count per test subject and time point was used.

### 2.8 Isolation of PBMCs

Isolation of peripheral blood mononuclear cells (PBMCs) from whole blood was performed by using 50 mL Leucosep tubes (Greiner Bio‐One) with 15 mL of Ficoll‐Paque TM PLUS (Cat# 17–1440‐02, GE Healthcare) (Sumbalova et al., 2020). Eighteen milliliter of blood was diluted in the same amount of RPMI‐1640 medium (#A10491‐01, Gibco Thermo Fisher Scientific) and centrifuged at 1000 g for 10 min (acceleration 6, no brakes). The buffy coat (layer of leukocytes and platelets) was then collected and washed twice with RPMI‐1640 (120 g, 10 min, acceleration 9, brake 6) to reduce the PLT/PBMC ratio below 7 (Sumbalova et al., 2020). The buffy coat was resuspended in 0.5 mL RPMI‐1640 and the number of PBMCs and platelets was measured with Sysmex XP‐300 (Sysmex Corporation). Based on the cell number, the PBMC suspension was further diluted to RPMI‐1640 (Sigma‐Aldrich) to achieve a cell concentration of 2 × 10^6/ml.

### 2.9 Bioenergetic function of PBMCs

Measurement of bioenergetic function of intact PBMCs was performed with a high‐resolution respirometer (HRR, O2k FluoRespirometer, Oroboros Instruments) as single measurements. Several bioenergetic function parameters were analyzed by using the substrate‐uncoupler‐inhibitor titration 3 (SUIT3) protocol designed for intact cells (Doerrier & Gnaiger, 2003). Cell suspension was added to chambers of HRR, and after balancing, routine respiration (R) was measured. To inhibit ATP synthase and induce leak respiration (L), which illustrates proton leak and the part of cellular respiration not participating in ATP production, oligomycin (0.015 μM, Cat# O4876, Sigma–Aldrich) was injected. Next, uncoupler carbonyl cyanide m‐chlorophenyl hydrazone (CCCP) (0.25–2.0 μM, Cat# C2759, Sigma‐Aldrich), was titrated with optimal concentration to get maximal ET‐capacity. Finally, rotenone (0.5 μM, Cat# R8875, Sigma–Aldrich) and antimycin A (2.5 μM, Cat# A8674, Sigma–Aldrich) were injected to inhibit respiration complexes I and III function. This was done in order to measure residual oxygen consumption (ROX), which illustrates oxidative side reaction in cellular respiration after inhibition of the electron transfer pathway. ROX was subtracted from the cellular respiration parameters (Doerrier & Gnaiger, 2003). Measurements of bioenergetic function were performed at 37°C in RPMI‐1640 medium (Sigma‐Aldrich). The solubility factor for RPMI‐1640 was 0.890 at 37°C. DatLab software version 7.3.0.3 (Oroboros Instruments) was used for data collection, and absolute cellular respiration values were normalized for the cell number per chamber, determined by Sysmex XP‐300 (Sysmex Corporation).

Free routine activity (R‐L) and spare capacity (ET‐capacity–R) were calculated via the abovementioned cellular respiration parameters. In addition, several flux control ratios (FCRs) and coupling‐control efficiencies were calculated for internal normalization of mitochondrial respiration. FCRs show cellular respiration parameters in relation to a reference state whereas coupling‐control efficiencies illustrate changes in cellular respiration normalized to reference state (Gnaiger, 2020). FCRs including L/R coupling‐control ratio, L/E coupling‐control ratio, R/E control ratio, and net R/E control ratio, as well as coupling‐control efficiencies R‐L control efficiency ((R‐L)/R), E‐L coupling control ((E‐L)/E) and E‐R control efficiency ((E‐R)/E) were calculated by using routine respiration (R), leak respiration (L) and maximal ET‐capacity (Gnaiger, 2020).

Additionally, the bioenergetic health index (BHI), which illustrates the bioenergetic state of the cells and mitochondrial function, was calculated with the following equation (Chacko et al., 2014, 2016):

$$\frac{\mathrm{ATP}-\text{linked respiration}\times \text{spare capacity}}{\text{leak respiration}\times \text{non}-\text{mitochondrial respiration}}$$

### 2.10 Statistical analysis

Statistical analyses were performed by using the Statistical Package for Social Sciences (SPSS version 28 for Microsoft Windows, IBM). Summary statistics are reported as mean with standard deviation (*SD*). All outcome variables were inspected for outliers, which were excluded from the analysis (>3 × interquartile range [IQR]). A *p*‐value <0.050 was considered statistically significant in all analyses. When examining the difference between HIGH and LOW E2 groups in background characteristics, Student's samples *T*‐test was used. Repeated measures ANOVA was used to examine whether there were differences in the study variables between HIGH and LOW E2 groups or between time points (repeated factor: PRE, POST and 1 h POST exercise) or responses between groups over time (group × time interaction). Mauchly's test was applied for verifying the assumption of sphericity, and if it was violated, Greenhouse–Geisser correction was used. In pairwise comparisons, Bonferroni adjustment for multiple comparisons was applied. When a significant group × time interaction or a main effect of time was detected, within‐subject contrasts for adjacent time points were used as post hoc tests. To assess whether aerobic fitness level is affecting the difference in cellular bioenergetic function results between groups, V̇O_2peak_/lean body mass was added to the analyses as a covariate.

## 3 RESULTS

### 3.1 Background data

The participants in the HIGH E2 and LOW E2 groups were similar in age, BMI, and body fat percentage (Table 1). However, the participants in the LOW E2 group had higher V̇O_2peak_ both relative to body mass and lean body mass (*p* ≤ 0.043, Table 1). Hence, to control for the difference in aerobic fitness level in cellular bioenergetic function results between groups, V̇O_2peak_/lean body mass was used as a covariate. There were no differences in the self‐reported or accelerometer‐measured leisure‐time physical activity (*p* ≥ 0.736, Table 1).

### 3.2 White blood cell count

**FIGURE 1:**
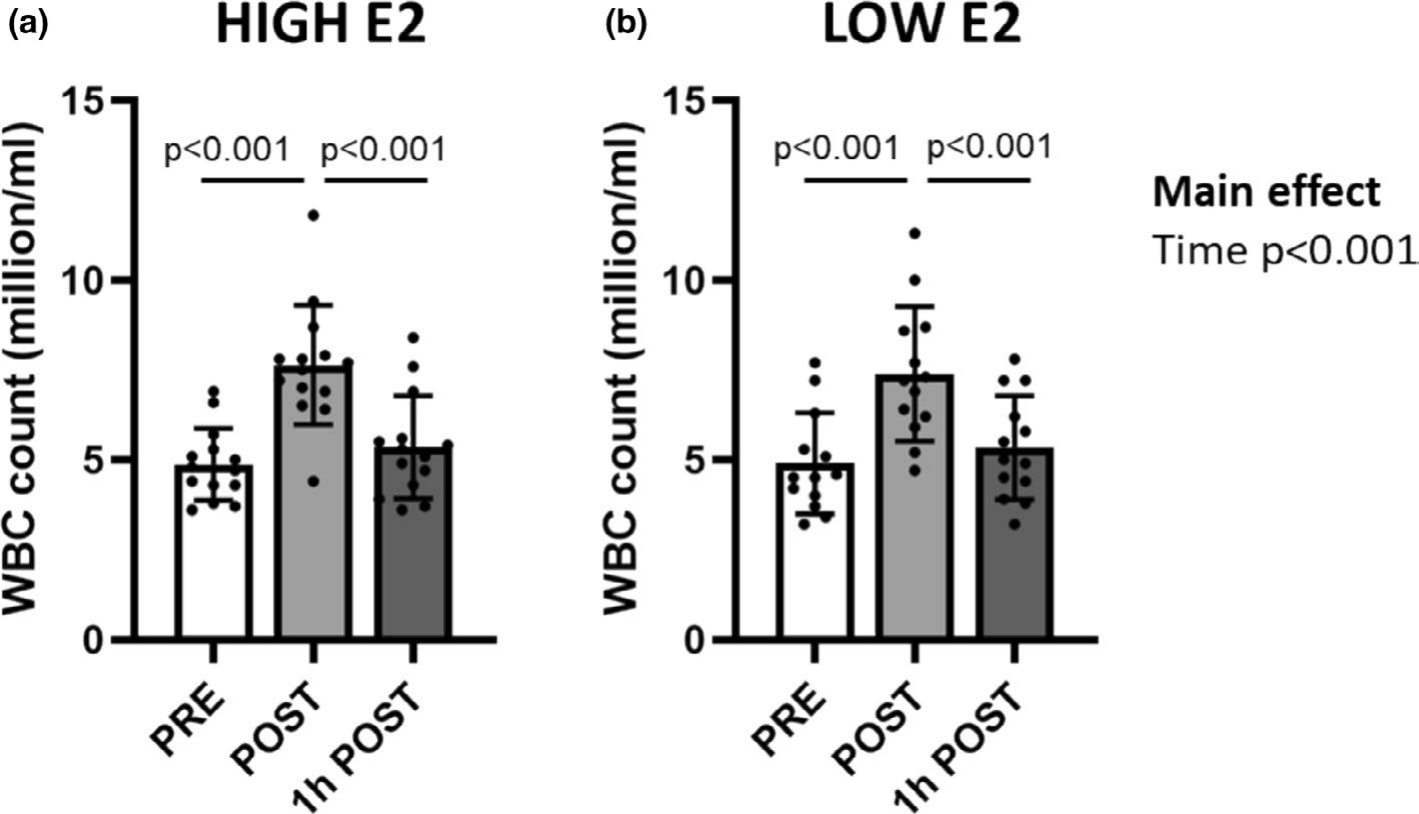
White blood cell (WBC) count (million/ml) in HIGH E2 (a) and LOW E2 (b) groups in time points PRE, POST and 1 h POST acute bout of exercise. Figures show individual data points with mean and standard deviation (*SD*). *n* = 12–14/group.

There was no group × time interaction or a main effect of group on WBC count (both *p* ≥ 0.522), but there was a significant main effect of time (*p* < 0.001, Figure 1). In both study groups, an acute bout of exercise increased the number of WBCs in blood (POST compared with PRE and 1 h POST, *p* ≤ 0.001, Figure 1). After adjusting for plasma volume shift, results still showed an increase in WBC count (HIGH E2 45.2% ± 26.3% and LOW E2 41.7% ± 28.3%), but there was no difference in the increase between the study groups (*p* = 0.743). Since the plasma volume shift did not affect WBC count, data was not adjusted for the plasma volume shift for the analysis (Figure 1, Table 2).

**TABLE 2:** Blood parameters of the study participants measured with an automated hematology analyzer.

Blood parameter	HIGH E2 (*n* = 14)			LOW E2 (*n* = 12)			*p* value		
	PRE	POST	1 h POST	PRE	POST	1 h POST	Group	Time	Group*time
Neutrophils (%)	54.34 (9.57)	51.96 (8.45)	64.85 (10.28)***, ###	52.16 (8.37)	48.54 (12.39)	60.60 (11.71)**, ###	0.342	**<0.001**	0.634
Lymphocytes (%)	35.63 (9.41)	39.43 (7.16)	27.76 (9.10)***, ###	38.37 (8.41)	41.80 (12.85)	31.97 (11.01)*, ###	0.386	**<0.001**	0.758
Monocytes, basophils, and eosinophils (%)	9.81 (2.28)	8.26 (2.55)	7.39 (2.39)**	9.48 (2.92)	8.42 (1.90)	7.43 (2.36)	0.852	**0.006**	0.516
Neutrophils (10^9/l)	2.70 (0.98)	4.04 (1.33)***	3.56 (1.42)**, #	2.51 (1.07)	3.54 (1.53)**	3.27 (1.46)*, #	0.472	**<0.001**	0.722
Lymphocytes (10^9/l)	1.69 (0.42)	2.96 (0.59)***	1.41 (0.28)**, ###	1.73 (0.31)	3.00 (0.93)***	1.53 (0.36)***, ###	0.824	**<0.001**	0.711
Monocytes, basophils, and eosinophils (10^9/l)	0.49 (0.11)	0.65 (0.18)**	0.39 (0.15)###	0.43 (0.16)	0.61 (0.21)**	0.38 (0.13)##	0.354	**<0.001**	0.370
Red blood cells (10^12/l)	4.18 (0.32)	4.60 (0.30)***	4.24 (0.31)##	4.54 (0.29)	4.87 (0.27)*	4.67 (0.22)	**0.003**	**<0.001**	**0.039**
Hemoglobin (g/l)	127 (8.46)	139 (6.78)***	129 (7.32)###	133 (7.42)	143 (6.24)**	137 (5.20)	**0.024**	**<0.001**	**0.031**
Hematocrit	0.38 (0.02)	0.42 (0.02)***	0.38 (0.02)##	0.40 (0.02)	0.43 (0.02)*	0.41 (0.01)	**0.005**	**<0.001**	**0.024**
Mean corpuscular volume (fl)	90.80 (2.67)	91.43 (2.59)***	90.69 (2.64)###	88.43 (2.18)	88.88 (2.30)**	88.33 (2.06)##	**0.018**	**<0.001**	0.564
Mean corpuscular hemoglobin (pg)	30.46 (1.37)	30.47 (1.39)	30.36 (1.29)	29.34 (0.65)	29.38 (0.74)	29.38 (0.75)	**0.021**	0.685	0.522

*Note*: Values show the mean with standard deviation (*SD*). *Compared with time point PRE. #Compared with time point POST. *p ≤ 0.050, **p ≤ 0.010, ***p ≤ 0.001. #*p* ≤ 0.050, ##*p* ≤ 0.010, ###*p* ≤ 0.001. Values with *p* < 0.050 are marked in bold.

We observed an increase in the percentage of neutrophils and a decrease in the percentage of lymphocytes when comparing 1 h POST to PRE and POST (all *p* ≤ 0.006, effect of time, Table 2). Quantitative analysis of cell type concentrations revealed a statistically significant increase across all examined cell categories immediately after exercise (POST vs. PRE) and a decrease 1 h POST exercise (1 h POST vs. POST, all *p* < 0.001, effect of time, Table 2).

For the number of red blood cells, hemoglobin, and hematocrit a significant group × time interaction was detected (*p* ≤ 0.039, Table 2). The observed interactions remained significant after adjusting for V̇O_2peak_/lean body mass (*p* ≤ 0.021, data not shown).

HIGH E2 group had a lower number of red blood cells and lower hemoglobin and hematocrit than LOW E2 group (*p* ≤ 0.024, main effect of group, Table 2). HIGH E2 group also exhibited a larger mean corpuscular volume and mean corpuscular hemoglobin than LOW E2 group (*p* ≤ 0.021, main effect of group, Table 2). When V̇O_2peak_/lean body mass was added as a covariate to the model, the difference between groups in the number of red blood cells, hemoglobin, and hematocrit remained significant (main effect of group *p* ≤ 0.049, data not shown).

**FIGURE 2:**
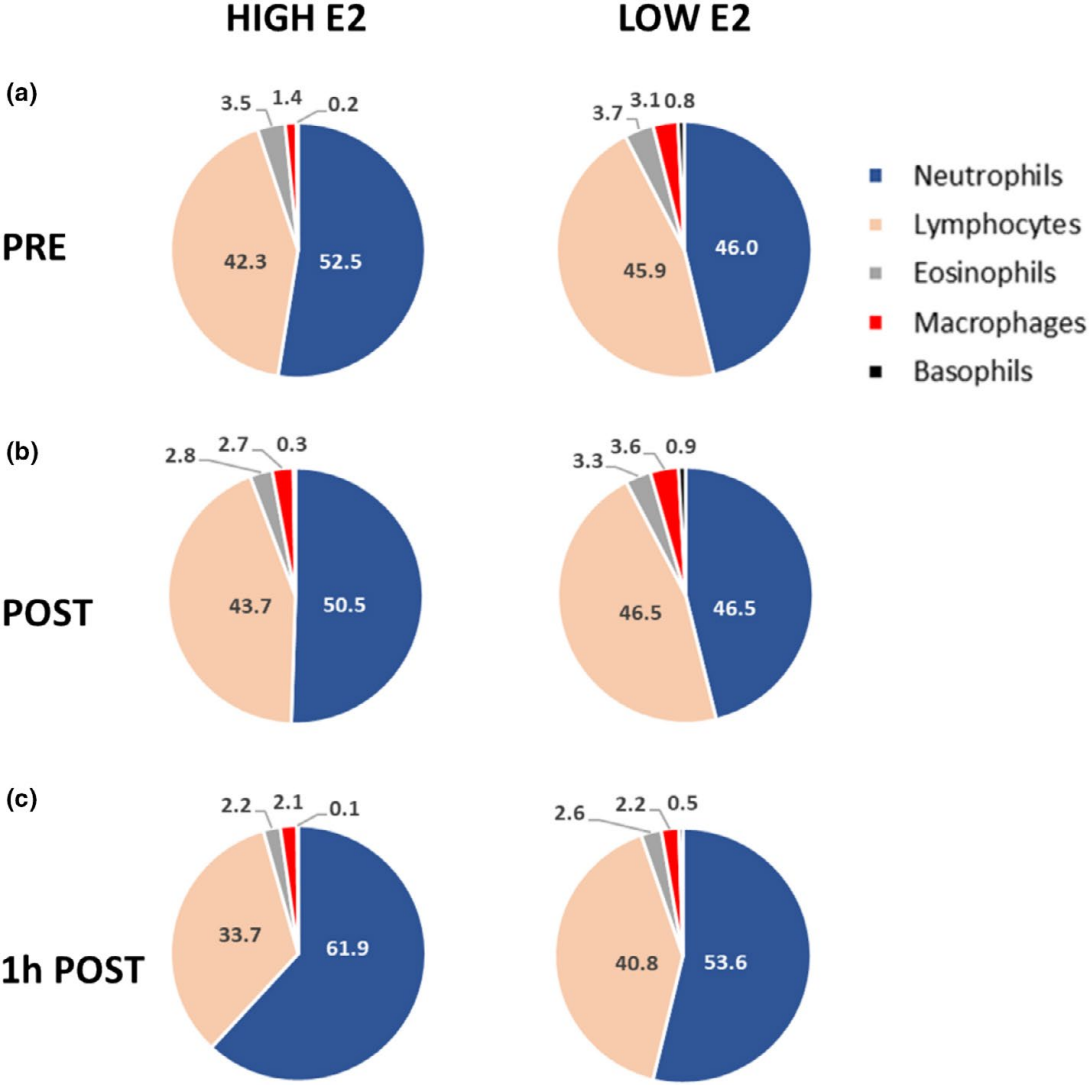
Differential white blood cell (WBC) count from stained blood samples in HIGH E2 and LOW E2 groups in timepoints PRE (a), POST (b) and 1 h POST (c) acute bout of exercise. Figures show relative percentage of neutrophils, lymphocytes, eosinophils, monocytes, and basophils. *n* = 12–14/group.

We further analyzed the percentage of neutrophils, lymphocytes, eosinophils, monocytes, and basophils separately via differential WBC count from stained blood samples (Figure 2). The measurements done by staining and the hematology automated analyzer had a high correlation in the percentage of neutrophils (*r* = 0.773, *p* < 0.001) and the percentage of lymphocytes (*r* = 0.788, *p* < 0.001) and were modest in the percentage of monocytes, basophils, and eosinophils combined (*r* = 0.367, *p* = 0.001).

### 3.3 Bioenergetics of PBMCs

**FIGURE 3:**
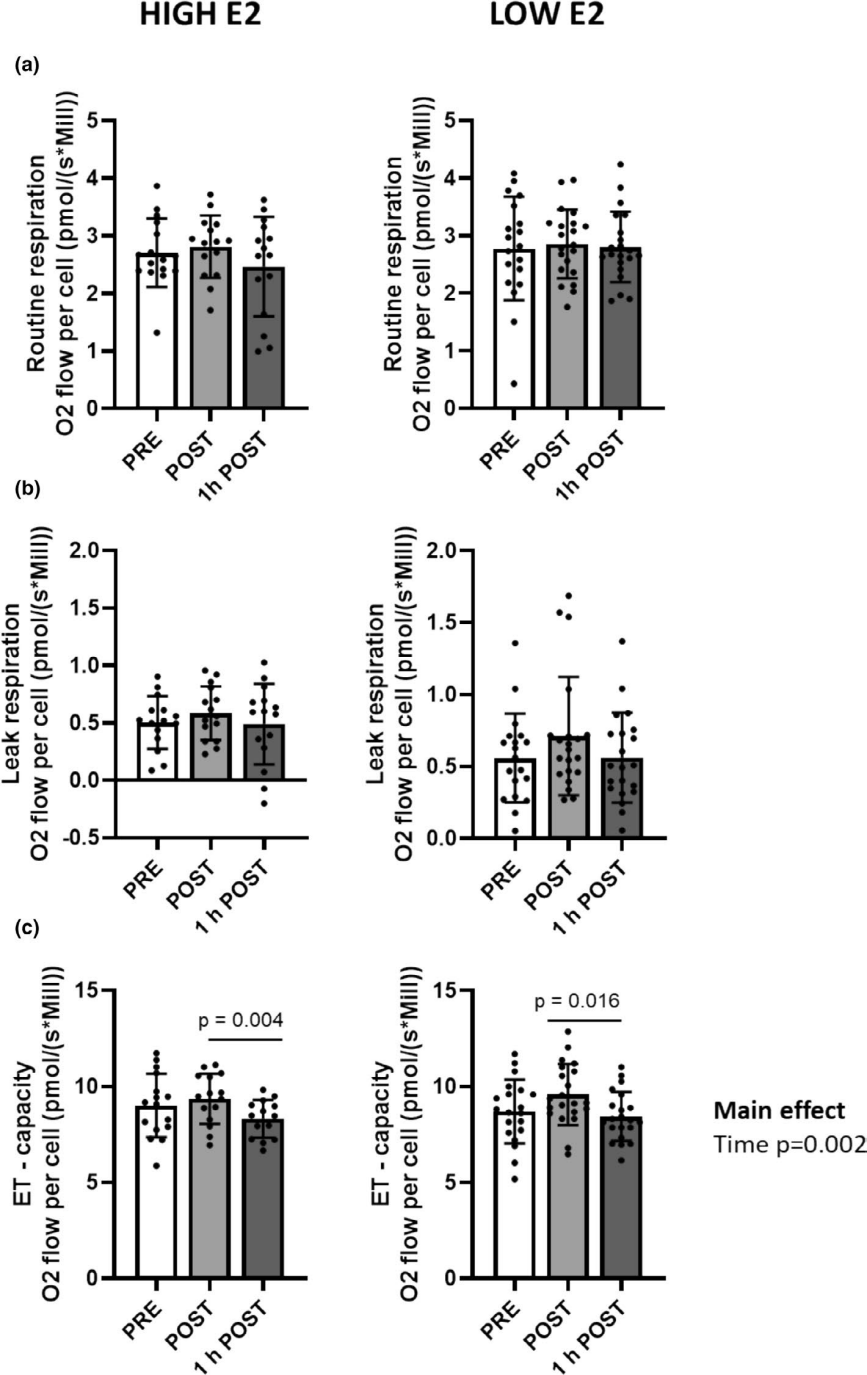
Bioenergetic function of PBMCs in HIGH and LOW E2 groups at time points PRE, POST, and 1 h POST. Figures represent routine respiration (a), leak respiration (b), and ET‐capacity (c) of PBMCs. Figures show individual data points with mean and standard deviation. *n* = 15–21/group.

When examining PBMC bioenergetic function, we found no group × time interaction or a main effect of group on routine respiration, leak respiration, or ET‐capacity (all *p* ≥ 0.265, Figure 3). However, we observed a main effect of time on ET‐capacity (*p* = 0.002) and a significant decrease in ET‐capacity at time point 1 h POST compared with POST in both HIGH and LOW E2 groups (Figure 3c). Flux control ratios, coupling control efficiencies, and bioenergetic health index of PBMCs are presented in Figures S1–S3 (no significant differences).

**FIGURE 4:**
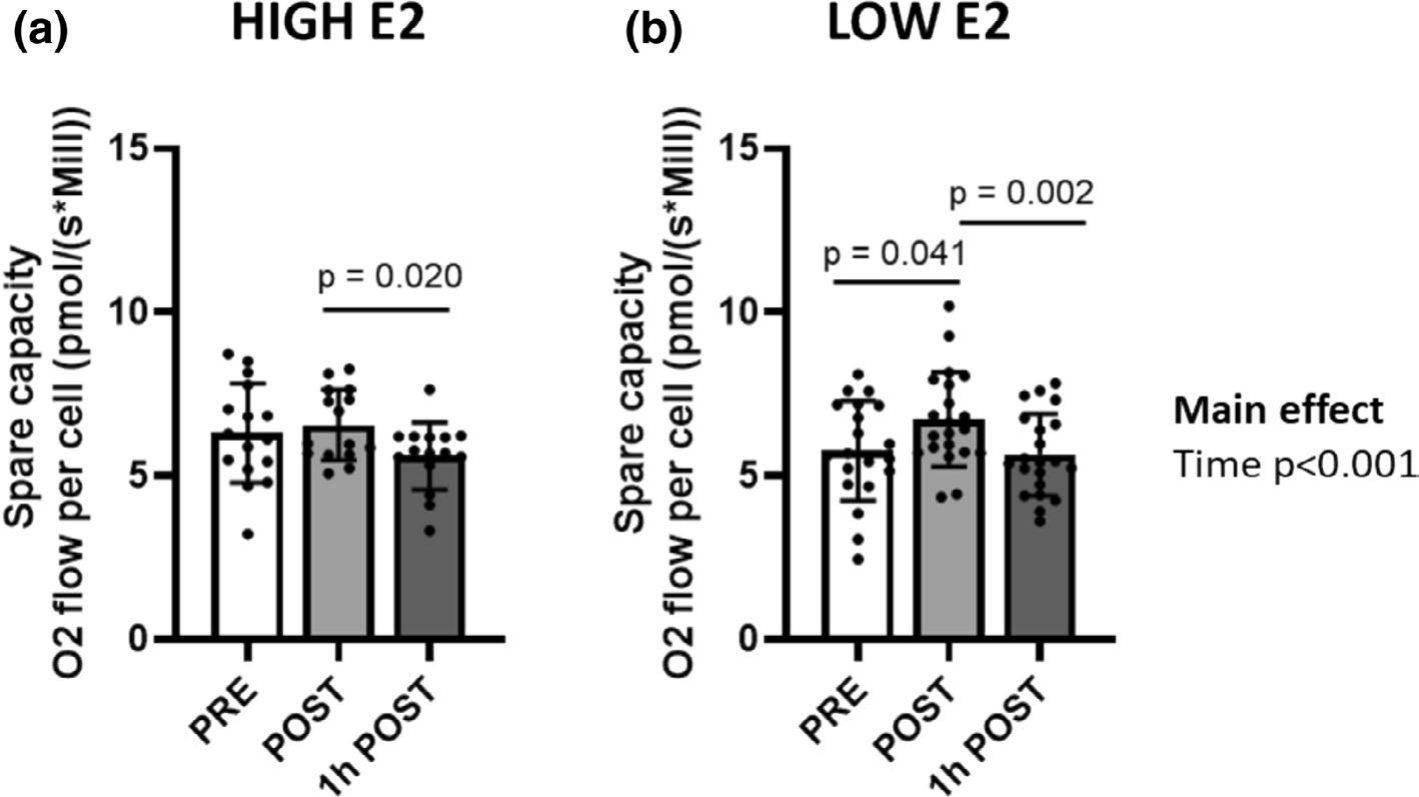
Spare capacity of PBMCs at different time points in HIGH (a) and LOW E2 (b) groups at time points PRE, POST, and 1 h POST. Figures show individual data points with mean and standard deviation (*SD*). *n* = 15–21/group.

When investigating spare capacity, we found no group × time interaction or a main effect of group (both *p* ≥ 0.215), but a main effect of time (*p* < 0.001, Figure 4). The spare capacity was lower at time point 1 h POST compared with POST (*p* < 0.020, Figure 4a). In the LOW E2 group, the spare capacity was higher immediately after an acute bout of exercise (POST) compared with time points PRE and 1 h POST (*p* < 0.050, Figure 4b).

**FIGURE 5:**
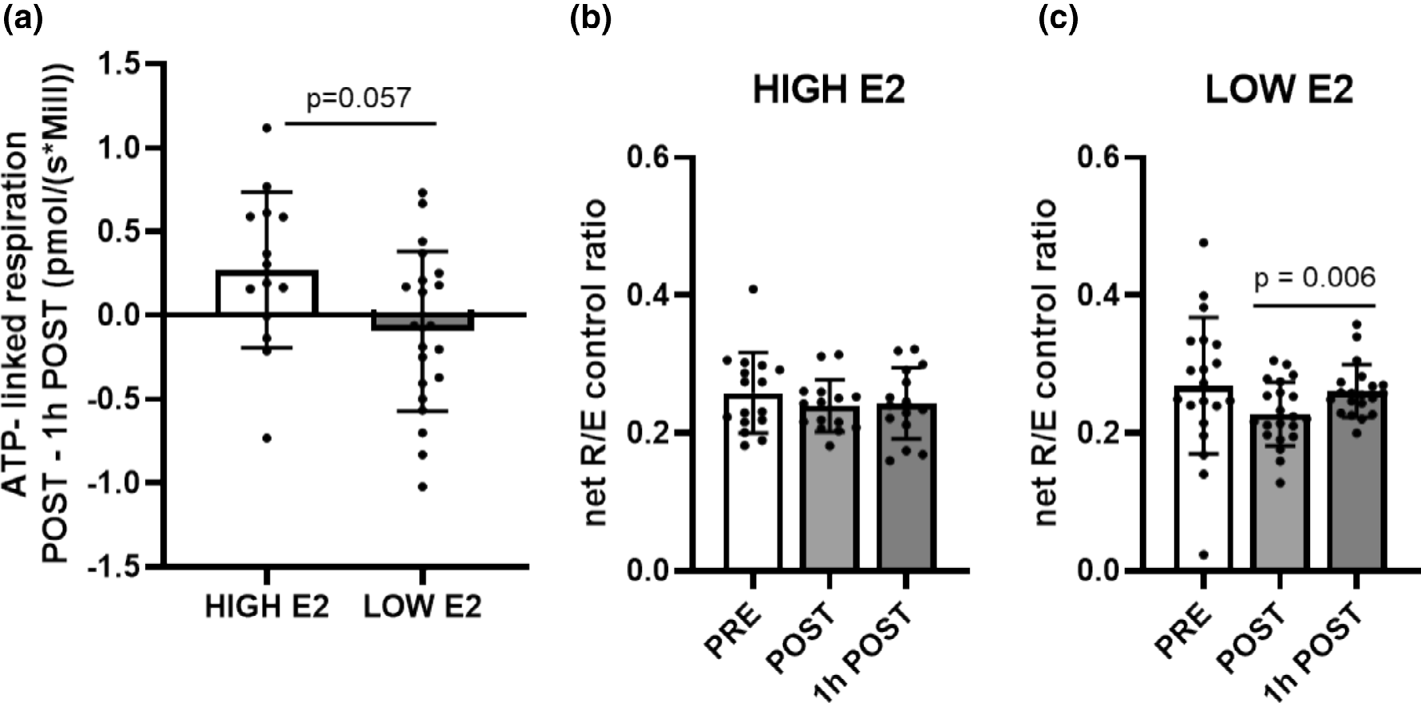
Difference in ATP‐linked respiration of PBMCs between HIGH and LOW E2 groups (a) and net R/E control ratio at time points PRE, POST, and 1 h POST in HIGH E2 (b) and LOW E2 group (c). Figure represents difference (POST–1 h POST) in ATP‐linked respiration between groups. Figures show individual data points with mean and standard deviation (*SD*). *n* = 15–21/group.

We observed no significant group × time interaction in ATP‐linked respiration or net R/E control ratio (both *p* ≥ 0.185). Also, there were no main effects of group or time on ATP‐linked respiration (difference between time points POST– 1 h POST) or net R/E control ratio (*p* ≥ 0.082). Even though group × time interactions were not statistically significant, visual inspection showed differential responses within a group. Therefore, we performed explorative comparisons between the groups, and ATP‐linked respiration showed a statistical difference between groups when POST and 1 h POST timepoints were compared. The ATP‐linked respiration was lower in LOW E2 than in HIGH E2 group before V̇O_2peak_ was added as a covariate (*p* = 0.028); yet, the difference remained close to significant also after adjusting for V̇O_2peak_ (*p* = 0.057, Figure 5a). In addition, in the LOW E2 group, the net R/E control ratio was higher at time point 1 h POST compared with POST (Figure 5b).

## 4 DISCUSSION

In the present study, we explored the link between systemic E2 levels and the bioenergetic function of PBMCs. Specifically, we investigated these associations both before and after an acute bout of endurance exercise. We observed that an acute bout of exercise increased the number of WBCs in blood in both HIGH and LOW E2 study groups. There was an increase in the percentage of neutrophils and a decrease in lymphocytes in response to exercise. However, no statistically significant associations were observed between systemic E2 level and PBMC bioenergetic function at the basal state or in the responses to acute exercise.

### 4.1 Acute bout of exercise induced a transient increase in white blood cell count independent of systemic E2 level

We observed an increase in the WBC count in both study groups in response to an acute bout of exercise. Previous literature has shown that the response of WBC count depends on the intensity and duration of the exercise (Cerqueira et al., 2019). Several studies have shown an increase in WBC count after a high‐intensity exercise (Connolly et al., 2004; de Gonzalo‐Calvo et al., 2015; Degerstrøm & Østerud, 2006; Nieman et al., 2012) (>64% of maximal oxygen consumption, VO_₂max_) supporting our findings. In our study, the WBC count returned to PRE level 1 h POST the acute bout of exercise; similarly, as observed in a study by Connolly et al. (2004) having the same time points (Connolly et al., 2004). However, also contradicting findings have been made: Nieman et al. (2012) showed that WBC count was still elevated 1 h post exercise (Nieman et al., 2012). The difference seen in this study may be due to the study population and exercise intensity and duration—study subjects were male cyclists, who performed ~2 h cycling at 60% of maximal work capacity, which differs from our female subjects having shorter and maximal exercise protocol. However, according to previous literature, the increase in WBC count in response to acute exercise is transient and returns to normal level within 48 h (Abbasi et al., 2014; de Gonzalo‐Calvo et al., 2015; Spiropoulos et al., 2010). As we observed no notable differences in the percentage of WBC types between time points PRE and POST, our results indicate that the acute increase in WBC count is due to a higher number of all WBC types, which is in line with previous findings (de Gonzalo‐Calvo et al., 2015; Nieman et al., 2012). However, we did observe an increase in the percentage of neutrophils and a decrease in the percentage of lymphocytes in response to exercise 1 h after testing. The increase in neutrophils with exercise is an established phenomenon that results from demargination from endothelial tissues, bone marrow and/or as part of the inflammatory response to exercise‐induced tissue damage (Peake, 2002; Pyne, 1994). Similar findings concerning lymphocytes were done by Connolly et al. (2004), showing a decrease in lymphocytes 1 h after acute exercise (Connolly et al., 2004). In addition, we showed that the number of red blood cells and platelets, as well as the level of hemoglobin and hematocrit, increased in response to an acute bout of exercise. The observed changes are due to the plasma volume shift, leading to an increase in the relative count of blood cells and concentration of hemoglobin (Matomäki et al., 2018).

HIGH E2 group had a lower number of red blood cells and lower hemoglobin and hematocrit than LOW E2 group after adjusting for V̇O_2peak_/lean body mass ratio. Even though E2 has not shown to directly impact these blood parameters, we have previously shown that postmenopausal women have higher red blood cell counts, hemoglobin concentrations, and hematocrits compared with menstruating premenopausal women (Kovanen et al., 2018), which is in line with the results reported here.

### 4.2 Systemic E2 level was not associated with the bioenergetic function of white blood cells in response to acute exercise

Present study showed no clear association between the systemic E2 level and PBMC bioenergetic function at the basal (PRE) state. In a previous study, sex‐associated differences in PBMC mitochondrial function were observed (Silaidos et al., 2018). Females exhibited higher levels of mitochondrial complexes I, I + II, and IV, as well as increased ET‐capacity and uncoupled respiration compared to males (Silaidos et al., 2018). Estrogens have been proposed to share a role in this observation. Indeed, several studies have demonstrated an estrogen‐induced upregulation of genes encoding for components of the mitochondrial electron transport chain, including complexes I and IV in vitro (Chen et al., 1998, 2003) and in vivo (Irwin et al., 2008; Nilsen et al., 2007). In addition, we have previously shown that E2 improves the mitochondrial bioenergetic function in skeletal muscle (Torres et al., 2018). According to previous studies and results obtained here, low estrogen level could be linked to decreased ATP production efficiency, yet the underlying mechanisms of the sex differences in mitochondrial function and the possible role of estrogens must be elucidated in more detail in the future.

We observed no clear change in ET‐capacity immediately after acute exercise (PRE vs. POST). A previous study by Liepinsh et al. (2020) observed an increase in PBMC respiration (routine, leak, and OXPHOS) in response to acute, low‐intensity endurance exercise in sedentary individuals (Liepinsh et al., 2020). Similarly, Stampley et al. (2023) reported an increase in routine respiration in swimmers after a maximal bout of exercise (Stampley et al., 2023). In contrast, Gatterer et al. (2018) found a decreased ET‐capacity following repeated‐sprint training in athletes (Gatterer et al., 2018). These studies suggest that both training status and exercise protocol influence the PBMC respiration response to exercise. Similarly to our findings, Theall et al. (2021) observed that PBMC bioenergetic function did not increase in response to acute exercise on a cell‐per‐cell basis, yet they showed an increase in mitochondrial oxidative capacity at the tissue level (Theall et al., 2021). Their study indicates that acute exercise preferentially mobilizes immune cells while concomitantly increasing PBMC mitochondrial oxidative capacity at the tissue level, rather than changing mitochondrial oxidative capacity at the cellular level (Theall et al., 2021). It is possible that acute exercise increased the oxidative capacity at the tissue level also in our study design, yet in this study we did not perform tissue‐level analysis. However, we report a significant decrease in ET‐capacity at the time point 1 h POST compared with POST in both study groups, indicating a reduced PBMC mitochondrial oxidative capacity following an acute bout of exercise. At basal state, lymphocytes have an oxidative nature while monocytes can switch between OXPHOS and anaerobic glycolysis (Chacko et al., 2013; Kramer et al., 2014). In addition to lower exercise‐induced stress at the 1 h POST time point, the reduced number of lymphocytes might partly explain the observed decrease in ETS.

While our main analysis did not indicate clear differences in changes in PBMC functional capacity based on E2 level, we performed explorative group comparisons, suggesting that E2 level may influence mitochondrial spare capacity, net R/E control ratio, and ATP‐linked respiration. These results, however, need to be interpreted with caution, as there was no significant group × time interaction, indicating similar behavior of the HIGH and LOW E2 groups. Mitochondrial spare capacity refers to the reserve capacity of mitochondria to produce ATP (Marchetti et al., 2020). Hence, our results suggest that the acute exercise stimulus may have a more proficient impact on the function of mitochondria at LOW E2 state. On the other hand, our results also suggested that the net R/E control ratio was lower immediately after the exercise compared with 1 h POST in the LOW E2 group. The net R/E control ratio compares phosphorylation‐related respiration to the rate of electron transport chain activity (Brand & Nicholls, 2011). Accordingly, a low net R/E control ratio suggests that the rate of electron transport activity is relatively slower compared to the rate of oxygen consumption and may indicate reduced ATP production efficiency (Hill et al., 2012). Our data may indicate that LOW E2 could be linked to decreased ATP production efficiency in response to acute exercise. This may suggest that in the LOW E2 group the acute exercise reduces ATP‐linked respiration. We have previously shown that estrogen deficiency caused by menopause blunts the systemic microRNA response to acute exercise (Karvinen et al., 2023). Further studies are warranted to examine the effect of these differences in the acute exercise response on exercise adaptations and health.

### 4.3 Study strengths and limitations

This study used serum FSH levels rather than relying solely on self‐report to accurately classify women as postmenopausal. This approach enabled a robust validation of menopausal status. The main limitation of our study is that the participants in the LOW E2 group had a higher V̇O_2peak_/lean body mass ratio, which was not explained by differences in the current leisure‐time physical activity levels. The HIGH E2 group contained women with both natural E2 production and women using exogenous E2, effects of which may differ. To overcome this issue, V̇O_2peak_/lean body mass was used as a covariate to control for the difference in aerobic fitness level in cellular bioenergetic function results between groups.

## 5 CONCLUSION

This study examined the associations between systemic E2 level and WBC count and PBMC bioenergetics in response to an acute bout of exercise. We show that an acute bout of exercise induced a transient increase in WBC count independent of systemic E2 level. No statistically significant associations were observed between systemic E2 level and PBMC bioenergetic function at the basal state or in the responses to acute exercise. Future studies should utilize larger sample sizes to thoroughly assess the effect of systemic E2 level on acute exercise response and the possible effects on exercise adaptations and health of postmenopausal women.

## AUTHOR CONTRIBUTIONS

Conceptualization: EKL and ML; Project administration: EKL; Funding acquisition: EKL, SK, and ML; Resources: EKL and ML; Supervision: EKL, ML, and SK; Methodology: ML, SK, EL, H‐KJ, JEK, and BH; Data analysis: SK, EL, BH, JEK, and AK; Visualization: SK, EL, and BH; Writing–original draft: SK and EL; Writing–review and editing: all authors.

## FUNDING INFORMATION

This study was funded by grants from the Research Council of Finland (grant numbers #314181, #335249, and #309504 to EKL, grant numbers #332946 and #354603 to SK and grant number #341058 to ML).

## CONFLICT OF INTEREST STATEMENT

The authors declare no conflicts of interest.

## ETHICS STATEMENT

The study was performed in accordance with the Declaration of Helsinki. All participants provided written informed consent, and the study was approved by the ethical committee of the Central Finland Health Care District (9 U/2018).

## Supporting information


Figures S1–S3.

## Data Availability

All data are presented in the article. Data are available upon reasonable request from the authors.
