# Supplementary material for: The acute exercise response of peripheral blood mononuclear cells and their bioenergetic function in women with high and low systemic estradiol levels
Source: Physiol Rep. 2025 May 1;13(9):e70296. doi: 10.14814/phy2.70296 (PMC12045702; doi:10.14814/phy2.70296)
Supplement: Supplementary file 1 — Figures S1–S3. [file PHY2-13-e70296-s001.docx]

## Supplementary material


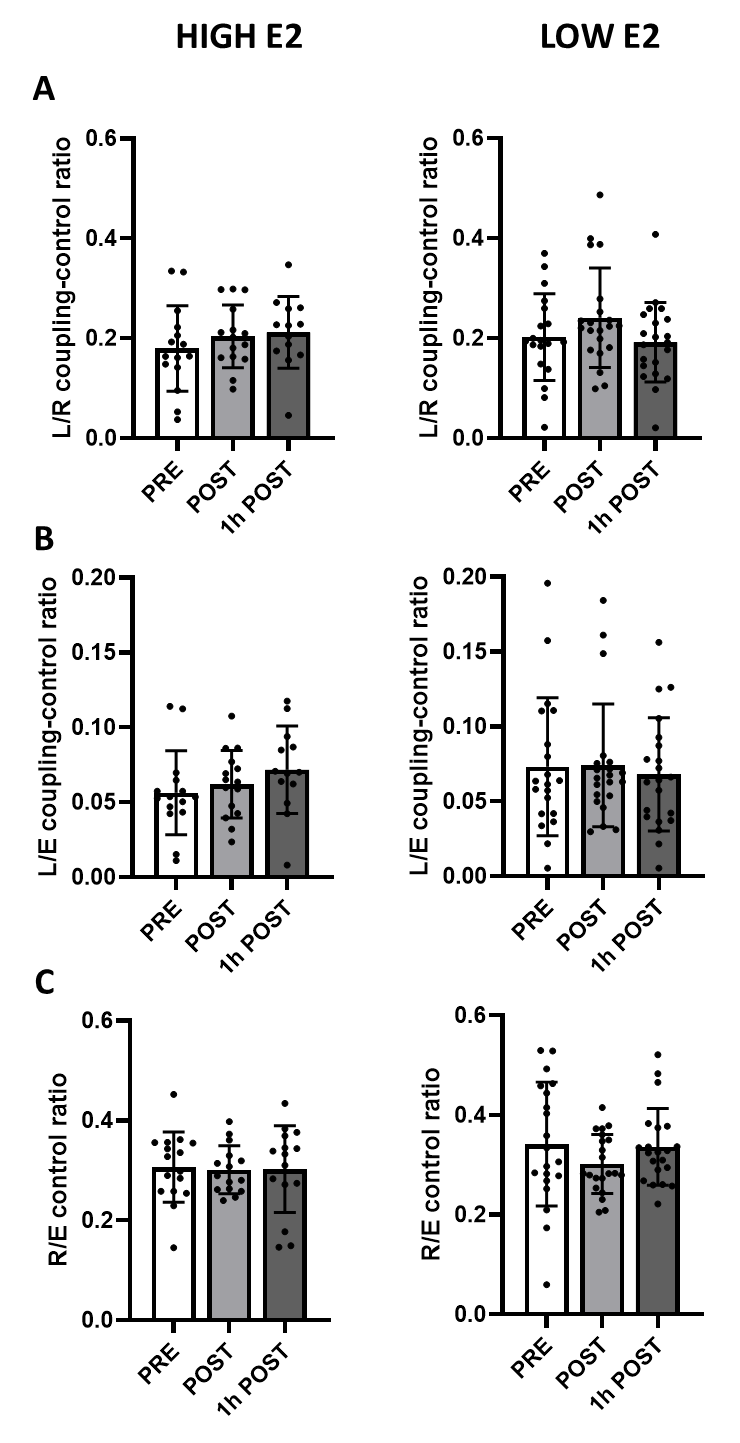


**Supplementary Figure 1**. Flux control ratios of PBMCs in HIGH and LOW E2 groups at time points PRE, POST and 1h POST. Figures represent L/R coupling-control ratio (**A**), L/E coupling-control ratio (**B**), and R/E control ratio of PBMCs (**C**). Figures show individual data points with mean and standard deviation (SD).


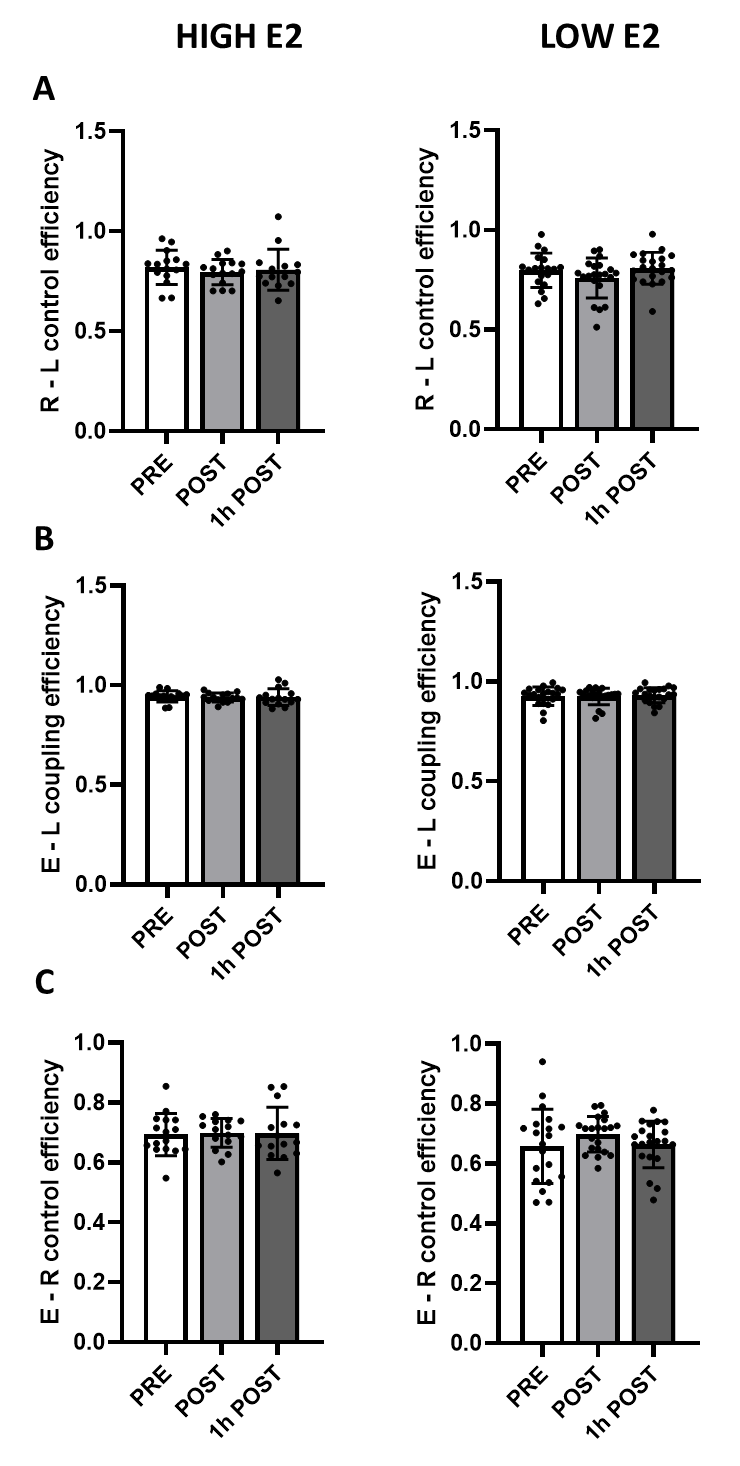


**Supplementary Figure 2**. Coupling control efficiencies of PBMCs in HIGH and LOW E2 groups at time points PRE, POST and 1h POST. Figures represent R-L control efficiency (**A**), E-L coupling efficiency (**B**), and E-R control efficiency (**C**) of PBMCs. Figures show individual data points with mean and standard deviation SD.

**Supplementary Figure 3.** Bioenergetic health index (BHI) of PBMCs in HIGH (**A**) and LOW E2 (**B**) groups at time points PRE, POST and 1h POST. Figures show individual data points with mean and standard deviation SD.

**HIGH E2**

**LOW E2**

**A**

**B**
